# Scientific American

**Published:** 1875-06

**Authors:** 


*Scientific American.*

We are pleased to welcome the re-appearance of the cheerful face of this scientific weekly upon our table. For solid, practical information it is unequaled, and will be found a welcome visitor in the homes of physicians. Munn & Co., publishers, No. 37 Park Row, New York. Price, $3 per annum.

